# Supplementary material for: Influenza A Virus Infection Reactivates Human Endogenous Retroviruses Associated with Modulation of Antiviral Immunity
Source: Viruses. 2022 Jul 21;14(7):1591. doi: 10.3390/v14071591 (PMC9320126; doi:10.3390/v14071591)
Supplement: Supplementary file 1 [file viruses-14-01591-s001.zip › MotifAnalysis.zip!/MotifOutput_PR8M/knownResults.html]

MotifOutput/ - Homer Known Motif Enrichment Results


# Homer Known Motif Enrichment Results (MotifOutput/)

Homer *de novo* Motif Results  
Gene Ontology Enrichment Results  
Known Motif Enrichment Results (txt file)  
Total Target Sequences = 23, Total Background Sequences = 45809

|  |  |  |  |  |  |  |  |  |  |  |  |
| --- | --- | --- | --- | --- | --- | --- | --- | --- | --- | --- | --- |
| Rank | Motif | Name | P-value | log P-pvalue | q-value (Benjamini) | # Target Sequences with Motif | % of Targets Sequences with Motif | # Background Sequences with Motif | % of Background Sequences with Motif | Motif File | SVG |
| 1 | T C G A C T A G A G T C A G T C C G T A C G T A A C G T T A G C T C A G T A C G | NFY(CCAAT)/Promoter/Homer | 1e-5 | -1.232e+01 | 0.0020 | 9.0 | 39.13% | 2803.5 | 6.12% | motif file (matrix) | svg |
| 2 | G A C T C G A T C A T G G C T A G C A T C G T A A G T C G C T A G C A T C G A T T A C G G C A T C G T A C A G T G A T C | DMRT6(DM)/Testis-DMRT6-ChIP-Seq(GSE60440)/Homer | 1e-4 | -1.005e+01 | 0.0095 | 5.0 | 21.74% | 805.2 | 1.76% | motif file (matrix) | svg |
| 3 | A T G C G A C T A C G T C T A G A C G T A C G T A C G T C T G A G A T C G C T A A G C T C G T A | Foxa2(Forkhead)/Liver-Foxa2-ChIP-Seq(GSE25694)/Homer | 1e-4 | -9.648e+00 | 0.0095 | 8.0 | 34.78% | 2978.8 | 6.50% | motif file (matrix) | svg |
| 4 | C G T A G C T A C G A T C T A G A C G T G T C A C G T A C G T A A G T C C G T A T G C A T A C G | FoxL2(Forkhead)/Ovary-FoxL2-ChIP-Seq(GSE60858)/Homer | 1e-4 | -9.263e+00 | 0.0104 | 8.0 | 34.78% | 3145.7 | 6.87% | motif file (matrix) | svg |
| 5 | A G C T A G T C A G T C A C G T C T A G A C G T A C G T A C G T C G T A A G T C G A T C C G T A | FOXP1(Forkhead)/H9-FOXP1-ChIP-Seq(GSE31006)/Homer | 1e-3 | -9.089e+00 | 0.0104 | 6.0 | 26.09% | 1608.6 | 3.51% | motif file (matrix) | svg |
| 6 | G C A T C G A T C T A G G C T A G C A T C G T A G A T C G C T A C G A T C G A T C T A G C G A T C G T A C G A T G A T C | DMRT1(DM)/Testis-DMRT1-ChIP-Seq(GSE64892)/Homer | 1e-3 | -8.943e+00 | 0.0104 | 5.0 | 21.74% | 1018.9 | 2.22% | motif file (matrix) | svg |
| 7 | C G A T A C G T C G T A C G T A A C G T A G C T T G C A C T G A C G T A A C G T A C G T C G T A | Phox2b(Homeobox)/CLBGA-PHOX2B-ChIP-Seq(GSE90683)/Homer | 1e-3 | -8.212e+00 | 0.0171 | 4.0 | 17.39% | 639.8 | 1.40% | motif file (matrix) | svg |
| 8 | G C T A T C G A C G T A C T A G A G C T G T C A G T C A C G T A A G T C C G T A | FOXA1(Forkhead)/MCF7-FOXA1-ChIP-Seq(GSE26831)/Homer | 1e-3 | -7.899e+00 | 0.0204 | 8.0 | 34.78% | 3829.0 | 8.36% | motif file (matrix) | svg |
| 9 | T A C G T A G C G C T A C G A T C T A G A C G T C A G T C A G T G C T A A G T C G T C A G C A T | FOXK2(Forkhead)/U2OS-FOXK2-ChIP-Seq(E-MTAB-2204)/Homer | 1e-3 | -7.122e+00 | 0.0395 | 6.0 | 26.09% | 2320.3 | 5.07% | motif file (matrix) | svg |
| 10 | C G T A C T G A C G T A C T A G T C G A C T A G A C T G C G T A C G T A T A C G A G C T A T C G | SpiB(ETS)/OCILY3-SPIB-ChIP-Seq(GSE56857)/Homer | 1e-2 | -6.667e+00 | 0.0560 | 4.0 | 17.39% | 966.8 | 2.11% | motif file (matrix) | svg |
| 11 | G C T A T C G A C G T A C T A G A G C T G T C A G T C A C G T A A G T C C G T A | FOXA1(Forkhead)/LNCAP-FOXA1-ChIP-Seq(GSE27824)/Homer | 1e-2 | -6.650e+00 | 0.0560 | 8.0 | 34.78% | 4615.4 | 10.08% | motif file (matrix) | svg |
| 12 | C T A G A G T C T A C G T A C G T G A C C G T A A C T G T A G C G C A T C A T G A T G C A G C T | Ascl1(bHLH)/NeuralTubes-Ascl1-ChIP-Seq(GSE55840)/Homer | 1e-2 | -6.647e+00 | 0.0560 | 10.0 | 43.48% | 7099.6 | 15.50% | motif file (matrix) | svg |
| 13 | C G A T C T A G A C G T G T C A C G T A C G T A A G T C C G T A | Foxo3(Forkhead)/U2OS-Foxo3-ChIP-Seq(E-MTAB-2701)/Homer | 1e-2 | -6.297e+00 | 0.0624 | 6.0 | 26.09% | 2721.1 | 5.94% | motif file (matrix) | svg |
| 14 | G A T C G C A T C T G A C G T A A G C T A G C T C G T A T C G A C T G A A C G T G C A T C G T A | Phox2a(Homeobox)/Neuron-Phox2a-ChIP-Seq(GSE31456)/Homer | 1e-2 | -5.960e+00 | 0.0811 | 4.0 | 17.39% | 1173.6 | 2.56% | motif file (matrix) | svg |
| 15 | A T G C T A C G A G T C G A C T T G C A C T G A G A C T A C G T C T G A T C A G | LHX9(Homeobox)/Hct116-LHX9.V5-ChIP-Seq(GSE116822)/Homer | 1e-2 | -5.533e+00 | 0.1159 | 8.0 | 34.78% | 5493.8 | 12.00% | motif file (matrix) | svg |
| 16 | G A T C G C T A C A G T A C G T T A C G A G T C A T G C C T A G A G T C T C G A | Zfp57(Zf)/H1-ZFP57.HA-ChIP-Seq(GSE115387)/Homer | 1e-2 | -5.464e+00 | 0.1165 | 3.0 | 13.04% | 658.2 | 1.44% | motif file (matrix) | svg |
| 17 | T C A G T G A C G T A C C G T A A C G T T G A C A C G T T C A G A G C T G A C T | NeuroD1(bHLH)/Islet-NeuroD1-ChIP-Seq(GSE30298)/Homer | 1e-2 | -5.322e+00 | 0.1263 | 6.0 | 26.09% | 3305.3 | 7.22% | motif file (matrix) | svg |
| 18 | T G A C A G T C C G T A A C T G G T A C A C G T A C T G A C G T G A C T G A T C | Twist2(bHLH)/Myoblast-Twist2.Ty1-ChIP-Seq(GSE127998)/Homer | 1e-2 | -5.159e+00 | 0.1406 | 9.0 | 39.13% | 7186.2 | 15.69% | motif file (matrix) | svg |
| 19 | G C A T G C A T C T G A A C G T C T G A A C G T C G T A C G T A C G T A A G T C G T C A G T C A | Foxf1(Forkhead)/Lung-Foxf1-ChIP-Seq(GSE77951)/Homer | 1e-2 | -5.127e+00 | 0.1406 | 6.0 | 26.09% | 3441.0 | 7.51% | motif file (matrix) | svg |
| 20 | T C A G C T A G A T G C G A C T T G C A C G T A A G C T A C G T C T G A T A C G | En1(Homeobox)/SUM149-EN1-ChIP-Seq(GSE120957)/Homer | 1e-2 | -5.068e+00 | 0.1406 | 9.0 | 39.13% | 7283.2 | 15.90% | motif file (matrix) | svg |
| 21 | C T A G A T C G G A C T A G T C A G T C A G T C G A C T C T G A A C T G C T A G A C T G C T G A | EBF(EBF)/proBcell-EBF-ChIP-Seq(GSE21978)/Homer | 1e-2 | -4.794e+00 | 0.1735 | 3.0 | 13.04% | 839.7 | 1.83% | motif file (matrix) | svg |
